# A Case of Breast Cancer With Ulceration of a Reconstructed Nipple Due to Local Recurrence

**DOI:** 10.7759/cureus.39563

**Published:** 2023-05-27

**Authors:** Tsuyoshi Nakagawa, Hiroki Mori, Noriko Uemura, Iichiro Onishi, Goshi Oda

**Affiliations:** 1 Breast Surgery, Tokyo Medical and Dental University, Tokyo, JPN; 2 Plastic Surgery, Tokyo Medical and Dental University, Tokyo, JPN; 3 Pathology, Tokyo Medical and Dental University, Tokyo, JPN

**Keywords:** nipple ulceration, deep inferior epigastric perforator flap, nipple reconstruction, local recurrence, breast cancer

## Abstract

Ulceration of a reconstructed nipple occurred in a woman in her 50s who had undergone mastectomy, axillary lymph node dissection, and deep inferior epigastric artery perforator flap reconstruction for right breast cancer. The implanted cartilage was removed on suspicion of infection and the ulcer was biopsied. Local recurrence was identified on histopathological examination. Local recurrence near a reconstructed nipple can cause ulceration because of the fragility of the reconstructed nipple tissue. If erosion or ulceration develops in the reconstructed nipple relatively long after surgery, pathological examination is warranted.

## Introduction

Breast diseases that cause erosions or ulcerative lesions on the nipple include Paget's disease and adenomas, as well as breast cancer. Diagnosis is not usually difficult if a biopsy is reliably performed. We report here a case in which ulcer developed on a reconstructed nipple after secondary breast reconstruction and local recurrence was later diagnosed. In cases of ulceration or erosion of the reconstructed nipple, infection and inflammation of the implanted cartilage are key differential diagnoses, but pathological examination of the ulcerated area is also crucial.

## Case presentation

A woman in her 50s became aware of a right breast tumor in the lower-outer quadrant of the right breast. After examination, breast cancer was diagnosed. Her past medical history included rheumatoid arthritis for which she is currently being treated. Her family history included breast cancer in her mother and two maternal cousins, but she did not have a BRCA mutation. The patient underwent mastectomy and sentinel node biopsy, then a tissue expander was inserted. Since a 1-mm metastasis was observed in the sentinel node, axillary lymph node dissection was also performed. Pathological results showed invasive ductal carcinoma of the breast, T2N1M0. The diameter of invasion was 30 mm and lymph node metastasis was present in 1 of the 16 dissected lymph nodes. The tumor was estrogen receptor-positive, progesterone receptor-positive, c-erbB-2 (HER2)-negative, with a Ki67 index of 48.9%. Nuclear grade was 2. No vascular invasion was identified. All surgical margins were negative. The patient had been scheduled to receive chemotherapy as postoperative adjuvant therapy, but ended up declining, so only endocrine therapy was administered. 

**Figure 1:**
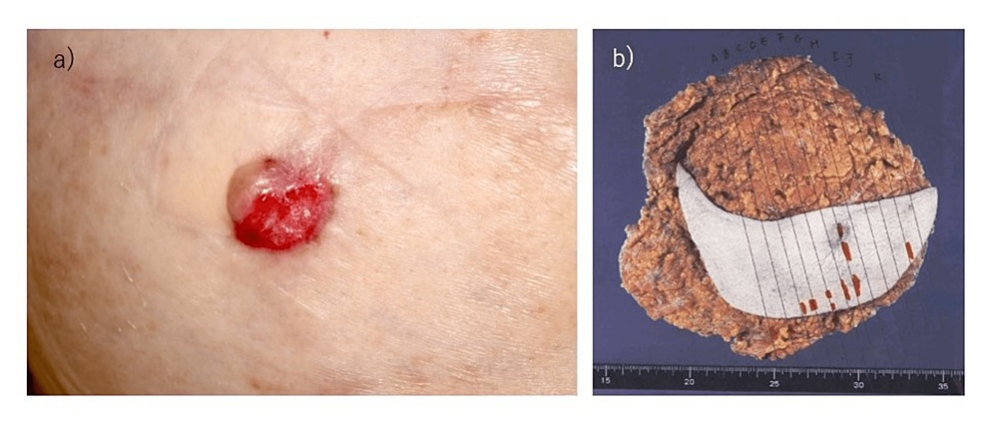
Reconstructed nipple and the resected flap. Ulceration of the reconstructed nipple (a) apparent in resected specimens of anterior chest skin and DIEP flaps. Red line shows localization of local recurrence within the resected flap (b) DIEP, deep inferior epigastric artery perforator

**Figure 2:**
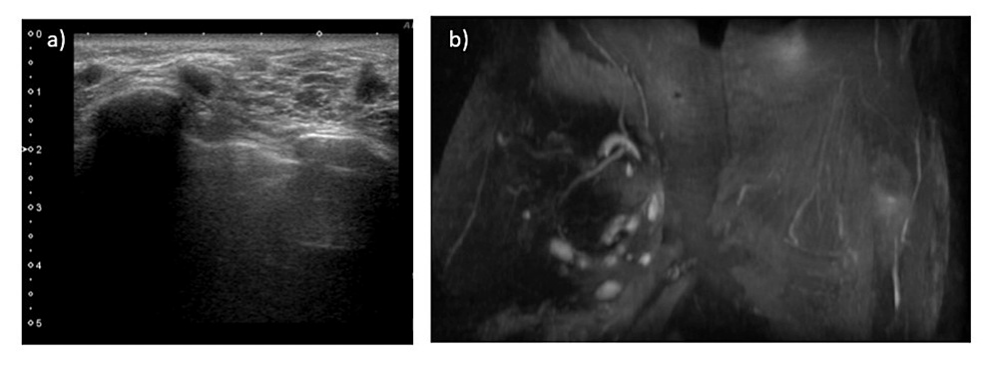
Imaging findings of local recurrences. Ultrasound examination (a) and MRI (b) of local recurrence under the skin. Ultrasonography shows multiple irregular, haloed masses, the largest of which is 12 mm in diameter, subcutaneously on the caudal side of the skin flap. MRI shows multiple subcutaneous irregular masses with contrast effect from the early phase.

**Figure 3:**
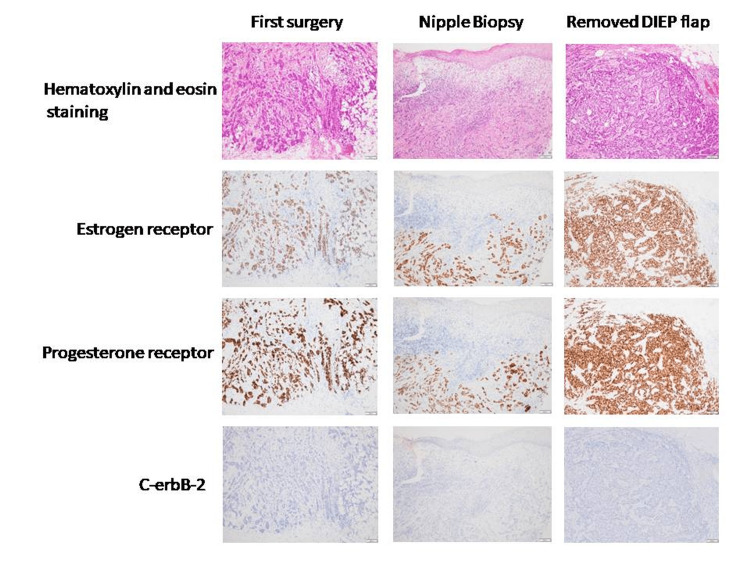
Pathological and immunostaining images of each specimen. Pathological and immunostaining images at first surgery, at nipple biopsy, and at resection of anterior chest skin and DIEP flap. DIEP, deep inferior epigastric artery perforator

Eight months after surgery, a two-stage breast reconstruction was performed with a deep inferior epigastric artery perforator (DIEP) flap. One year after surgery, nipple reconstruction was performed with the double opposing tab flap technique, and rib cartilage was implanted under the reconstructed nipple skin [1]. Two years postoperatively (1 year after nipple reconstruction), a 1-cm ulceration developed on the reconstructed nipple (Figure 1a). Although the ulceration improved with bucladesine sodium ointment application, inflammation of the cartilage implanted under the reconstructed nipple was considered likely and was therefore removed. Biopsy of the ulcerated area was performed. The biopsy results revealed adenocarcinoma, leading to a diagnosis of local recurrence of the previous breast cancer. No abnormalities were noted in the removed cartilage. When the right anterior breast (reconstructed breast) was examined, a 1-cm nodule was found under the skin of the caudal skin flap (Figure 2a,b). Needle biopsy of this area was therefore performed, revealing the nodule as adenocarcinoma and also showing multiple scattered local recurrences. At two years and three months postoperatively, the right anterior thoracic skin flap and reconstructed DIEP flap were removed. Multiple adenocarcinoma nodules up to 1 cm in diameter were observed in the resected specimen (Figure 1b). After this surgery, local treatment was prioritized, with chest wall irradiation followed by chemotherapy. Six years have passed since the first surgery, with no evidence of further local recurrences or distant metastases. Endocrine therapy remains ongoing. Pathological images of each specimen are shown in Figure 3. All pathological specimens represented invasive ductal breast carcinoma, as luminal-type scirrhous carcinoma with a high Ki67 index. 

## Discussion

Diseases that cause erosions and ulcers on the nipple include breast cancer, adenoma, and Paget's disease. When erosions or ulcers arise on a reconstructed nipple, inflammation is usually the first consideration because the lesions are on the skin. In this case, local recurrence was considered to have ulcerated the histologically fragile area of the reconstructed nipple. Complications for reconstructed nipples are difficult to discuss in general because of the variety of methods used for nipple reconstruction. Short-term complications include skin necrosis and infection, while long-term complications include a loss of cosmetic appearance due to reduced projection. Paolini et al. reported reconstructive nipple necrosis in 1%-29% of patients after nipple reconstruction and infection in 0.9%-16% [2]. In particular, reconstructive nipple necrosis was more common in a postoperative irradiation group compared to the non-irradiated group [3]. Further, local recurrence should be kept in mind for complications that occur a relatively long time after surgery. 

In this case, the resection margins were negative and recurrence through the puncture route was considered unlikely. Since lymph node metastasis was present at the time of the primary surgery, intralymphatic extension from the primary lesion into the subcutaneous lymphatics was assumed but not confirmed pathologically. Multiple local recurrences were concentrated under the skin of the caudal skin flap, so extension of the cancer into the lymphatic vessels seems the most likely possibility. Alternatively, unexpected seeding may have occurred during the primary surgery or secondary reconstructive surgery. 

Since the free skin flap can be used to repair defects in DIEP reconstruction, we avoid skin-sparing mastectomy in T2 patients and prefer mastectomy to remove sufficient skin directly above the tumor. In over 20 years of our own institution's experience, this was the only case in which removal of the whole reconstructed autologous flap was necessary due to local recurrence. Imaging and needle biopsy results before the primary surgery did not suggest an extremely high risk of local recurrence, and the course of the disease was difficult to predict. Postoperative chest wall irradiation was also not indicated. 

Postoperative adjuvant therapy prioritized local therapy, with irradiation to the chest wall first, followed by systemic chemotherapy. Since the decision to prioritize irradiation was made preoperatively, the patient had 2 months to start postoperative chemotherapy. As of 2.5 years since the surgery for local recurrence, no local recurrences or distant metastases have been identified. 

## Conclusions

We present a case in which ulceration appeared on the reconstructed nipple. Biopsy led to a diagnosis of local recurrence. Ulceration after a relatively long postoperative period should be considered a differential diagnosis for ulceration. 
